# From Macrohemodynamic to the Microcirculation

**DOI:** 10.1155/2013/892710

**Published:** 2013-02-27

**Authors:** Abele Donati, Roberta Domizi, Elisa Damiani, Erica Adrario, Paolo Pelaia, Can Ince

**Affiliations:** ^1^Sezione di Anestesia e Rianimazione, Dipartimento di Scienze Biomediche e Sanità Pubblica, Università Politecnica delle Marche, Ancona, Via Tronto 10, 60020 Torrette (Ancona), Italy; ^2^AOU Ospedali Riuniti, Via Conca 71, 60020 Ancona, Italy; ^3^Department of Translational Physiology, Academic Medical Center, University of Amsterdam, Meibergdreef 9, 1105 AZ Amsterdam, The Netherlands

## Abstract

ICU patients need a prompt normalization of macrohemodynamic parameters. Unfortunately, this optimization sometimes does not protect patients from organ failure development. Prevention or treatment of organ failure needs another target to be pursued: the microcirculatory restoration. Microcirculation is the ensemble of vessels of maximum 100 *μ*m in diameter. Nowadays the Sidestream Dark Field (SDF) imaging technique allows its bedside investigation and a recent round-table conference established the criteria for its evaluation. First, microcirculatory derangements have been studied in sepsis: they are mainly characterized by a reduction of vessel density, an alteration of flow, and a heterogeneous distribution of perfusion. Endothelial malfunction and glycocalyx rupture were proved to be the main reasons for the observed microthrombi, capillary leakage, leukocyte rolling, and rouleaux phenomenon, even if further studies are necessary for a better explanation. Therapeutic approaches targeting microcirculation are under investigation. Microcirculatory alterations have been recently demonstrated in other diseases such as hypovolemia and cardiac failure but this issue still needs to be explored. The aim of this paper is to gather the already known information, focus the reader's attention on the importance of microvascular physiopathology in critical illness, and prompt him to actively participate to achieve a more comprehensive understanding of the issue.

## 1. Introduction

The introduction in clinical practice of pulmonary artery catheter (PAC) about 40 years ago [1] allowed clinicians to measure the cardiac output (CO) at the bedside with the thermodilution technique [2]. Moreover, with an arterial and mixed venous gas analysis, arterial (CaO2) and mixed venous oxygen content (CvO2) could be easily calculated and oxygen availability (DO2) and consumption (VO2) consequently obtained by applying the following simple formulas: DO2 = CO∗CaO2 and VO2 = CO∗(CaO2 − CvO2). Old well-known physiologic data were available at the bedside as well as clinical parameters but their interpretation and utilization as a therapeutic target was and remains controversial to date.

Shoemaker was the first clinician to try to interpret and utilize these new hemodynamic data. He was a surgeon and monitored the high risk surgical patients with PAC before, during, and after the operations [3]. He observed that patients could be divided into three groups on the basis of outcome: survived, survived with complications, and died. From the analysis of the hemodynamic data, patients with better outcome resulted to have CO, DO2, and VO2 values higher than the others and, additionally, far higher than those considered as normal. Based on these observational data, he conceived the supernormal values of CO, DO2, and VO2 as therapeutic goals and obtained in his trial a reduction in mortality from 28% in the control groups to 4% in the protocol group [4]. Control groups included both patients with just a central venous catheter and patients monitored with a PAC, but with using normal values of CO, DO2, and VO2 as therapeutic targets. According to these data, physicians began to target supernormal CO, DO2, and VO2 values in critically ill patients, which seemed to be the best treatment. Nevertheless, the results were not so good. Gattinoni et al. did not find any difference between patients treated when targeting normal and supernormal CO, DO2, and VO2 values or a mixed venous saturation (SvO2) higher than 70% [5]. Hayes et al. found an increased mortality in patients treated with supernormal values as target [6]. Many doubts aroused among the intensivists, especially because of the hemodynamic stress due to hypervolemia and the infusion of inotropes, such as dobutamine, with an increased risk of myocardial ischemia and arrhythmias. That is why less than twenty years ago Vincent studied the VO2/DO2 relationship and observed that VO2 is usually independent from a wide range of DO2 because of compensation mechanisms [7]. At first, if CaO2 decreases, CO increases to maintain the same DO2 levels; then, when this compensation exhausted, another compensatory mechanism occurs due to increased oxygen extraction ratio (O2ER) which maintains normal levels of VO2 despite the DO2 reduction. When this compensation is exhausted too, VO2 becomes dependent on DO2 and the poorly efficient anaerobe metabolism begins. This leads to metabolic acidosis and oxygen debt. This normally happens when O2ER is near to 60%, but in some situations, for instance, during anaesthesia or sedation, critical O2ER could decrease until 30%. Therapy should aim to avoid the VO2/DO2 dependency to maintain O2ER lower than 30% near to normal values. In any case, according to the authors, only patients with high O2ER can benefit from hemodynamic optimization, while patients with normal O2ER, even if without high CO and DO2, do not need to be subjected to cardiovascular stress. The authors proposed the following simple dobutamine test: CO should be increased only if VO2 increases together with CO after dobutamine infusion, otherwise this is not necessary [8].

Despite these results in critically ill patients, in high risk surgical patients, the investigators continued to observe a decreased mortality using supernormal targets, including a recent meta-analysis [9–13]. Indeed some data indicate that also for these patients the VO2/DO2 relationship should be targeted and tested by the O2ER or the more feasible central venous oxygen saturation (ScvO2) [14]; however, these targets also seem to work only in high risk surgical patients and not in ICU patients. Why this discrepancy occurs?

Analyzing the Italian multicenter trial published in 1995 [5], in which ICU patients were treated following three hemodynamic goals (high values of CO, DO2, and VO2, normal values of the same ones; SvO2 > 70%) and enrolled within the first 48 hours from ICU admission. The difference between ICU and surgical patients is quite obvious: in surgical patients the moment when the “noxa patogena” begins (i.e., the operation) is exactly known and the hemodynamic treatment can be started at the same time or even before. In ICU patients it is almost always impossible to know when the pathogenic course of the illness begins, and even if that is known (i.e., trauma), ICU hemodynamic treatments are quite often started after several hours. Moreover, in the Italian study patients were enrolled even 48 hours after the ICU admission. Time is the issue. In 1995 Donati et al. published a study on a cardiac index (CI)/O2ER diagram [15]. CI and O2ER values were taken at the admission, after 12, 24, and 48 hours in any patient with a pulmonary catheter placed at the ICU admission, and values of each time point were plotted in a CI/O2ER diagram dividing survivors and nonsurvivors. Only at 24 h after ICU admission data were significantly differentiated between survivors and nonsurvivors, with survivors in the most favourable part of the diagram (normal/high CI and normal/low O2ER). Time is the issue. 

Rivers et al. more recently reported that septic shock patients who were aggressively treated, following a strict hemodynamic protocol and using the ScvO2 within the first 6 hours after hospital admission as therapeutic goal, had a better outcome than patients treated with normal target [16]. Nevertheless, some experts argued that the use of absolute goals themselves (i.e., ScO2, haemoglobin levels, central venous pressure, and mean arterial pressure) may not have been so crucial, while the positive results might mainly depend on the early implementation of the protocol and the greater promptness in the therapeutic approach. Once more, time is the issue [17].

Nowadays, the use of PAC has markedly decreased since new less invasive cardiac output measuring devices are available [18], such as PiCCO system, LiDCO system [19], EV1000/VolumeView system, the pressure analytical method (PRAM), and transthoracic or esophageal Doppler devices. However, whatever the monitoring method used, macrohemodynamic has to be optimized as soon as possible within the first hours from an initial hit. We can choose as therapeutic goals high CO, DO2, and VO2 values, according to Shoemaker's philosophy, or more “gentle” targets such as ScvO2 or O2ER [20], or fluid optimization following fluid-responsiveness parameters [21]. After these first hours, aiming to macrohemodynamic targets— although obviously important—is not sufficient to prevent organ failures or death, for which we need to identify some other targets. Treating the microcirculation might be the solution.

The microcirculation is the ensemble of vessels with diameter lower than 100 *μ*m and we can distinguish between small vessels (diameter lower than 20 *μ*m), medium vessels (diameter between 20 and 50 *μ*m), and great vessels (50–100 *μ*m).

**Figure 1:**
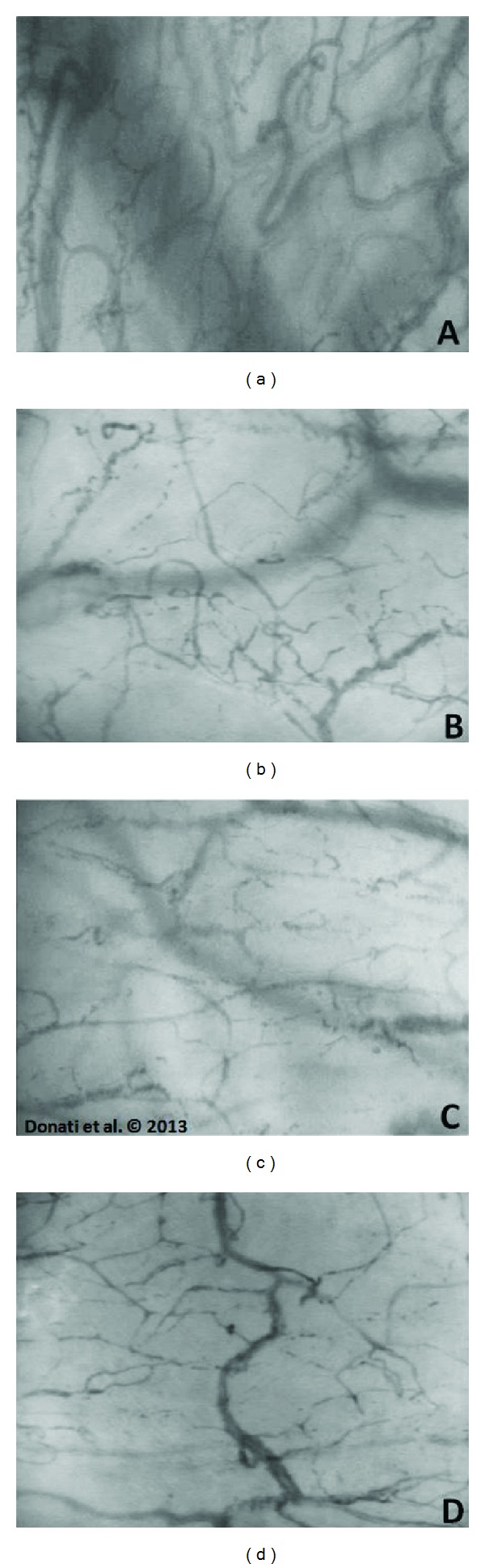
SDF images of the sublingual microcirculation. (a) Healthy subject; (b) septic shock; (c) hypovolemia; (d) cardiogenic shock.

Until few years ago, we were not able to observe the microcirculation at the bedside: indeed, intravital microscopy needs a back light and a circulating dye, conditions that could not be usually applied in the clinical practice. The Orthogonal Polarization Spectral (OPS) [22] and, more recently, the Sidestream Dark Field (SDF) imaging have allowed clinicians to observe in vivo, at the bedside, the sublingual microcirculation: this site is not only the most accessible location to examine, but it also has to be considered as an excellent mirror for the splanchnic microcirculation, as demonstrated by Boerma few years ago [23, 24].  Figure 1(a) provides an example of the sublingual microcirculation as it appears under physiological conditions.

In 2005, a round-table conference was organized in Amsterdam in order to score the microcirculation and the following parameters have been suggested: a measure of vessel density (total or perfused vessel density), two indices of vascular perfusion (proportion of perfused vessels and microcirculatory flow index), and a flow heterogeneity index (Table 1) [25].

These indices answer the three crucial questions that we should ask: how many vessels are perfused, what is the quality of the flow, and whether there are nonperfused areas next to the well-perfused ones.

But now the main question is which are the alterations we can find in different pathologies, such as sepsis, hypovolemia, or cardiac failure?

## 2. Microcirculation in Sepsis

Sepsis is the first pathophysiologic condition in which microcirculation was studied. Microcirculatory alterations in sepsis have been found in both experimental and human studies [26, 27]. 

Alterations are both quantitative, such as reduced vessel density, and qualitative, such as altered blood flow (slowed, intermittent, or even stopped). 

Moreover, heterogeneity of perfusion has been observed, with normally perfused areas bordering areas with altered capillary flow: the consequent increase in the distance between capillaries and cells makes hypoxia easier to quickly appear.

One of the main manifestations of heterogeneity is the appearance of areas with vascular stop flow and flow shunting from the arterial circulation to the venous, particularly in the intestinal villi, liver, diaphragm, skeletal muscle, and sublingual microcirculation.  Figure 1(b) shows an example of the sublingual microcirculation during sepsis. 

The PO_2_ gap can be used to quantify the oxygen extraction deficit that follows such shunt, representing a marker of severity of the shunt, and it is clinically associated with blood lactate and venous PO_2_ increase [26].

It has been hypothesized that these phenomena (heterogeneity, stoppage and shunting of the flow, perfusion deficit) may derive from a failure in autoregulation mechanisms, first of all due to an altered expression of the inducible nitric oxide synthase (iNOS) in some areas of the vascular bed; in regions where iNOS is poorly produced, vasodilation may be impeded up to the degree that it will not be sufficient to ensure the perfusion [28, 29]. 

**Figure 2:**
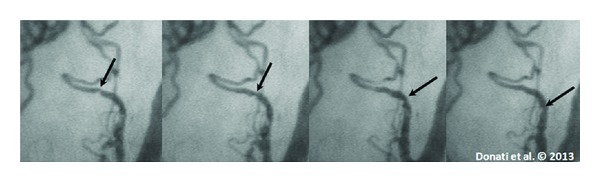
Sequence of SDF images of the sublingual microcirculation in a septic patient, showing the passage of a rolling leukocyte in a postcapillary venule.

There are many reasons for these alterations: microthrombi, capillary leakage, leukocyte rolling (Figure 2), and rouleaux phenomenon, but endothelial malfunction and glycocalyx ruptures probably play a central role.

It is well known that the endothelial cell is the crucial component of this auto-regulated system. Two endothelial functions among the others are the shear stress transduction and the activation of the response to catecholamines, prostaglandins, endothelin, bradykinin, thromboxane, and adenosine. It also participates to cell-to-cell communication in order to attend at local signs integration [30, 31].

Endotoxemia damages the endothelial cell, thereby breaking this chain and potentially impeding a sufficient tissue perfusion to be assured. 

The septic status is also associated with the reduction of the arteriolar muscular tone (with a lower response to adrenergic stimuli), impairment of red blood cells deformability (even more for the old ones), and increase in platelet aggregation tendency [30–33].

However, the exact reaction of platelets, red blood cells, and leucocytes is poorly understood.

Red blood cells become rigid and unshrinkable, haematic viscosity increases, and fibrin deposition rises. When, finally, platelets aggregate, microthrombi appear and drastically occlude smaller vessels [32, 33].

Bateman et al. observed that the leukocyte rolling, adhesion, and activation is an early step in septic evolution and it has to be attributed to an upregulation of adhesion molecules and inflammatory cascade [34]. 

The following radical oxygen species production may be considered, the main responsible as factor for the glycocalyx rupture. 

The glycocalyx represents a blood-tissue interface deriving from the endothelial cells and consisting of a proteoglycan, hyaluronan, and glycosaminoglycan-made layer, combined with plasmatic proteins. It participates to vascular tone regulation and mechanic impulses transduction and is also responsible for RBCs velocity variation. A rupture in its structure impairs all these mechanisms [34–36]. 

As already said, restoring a normal microcirculation is essential for a good outcome; therefore, this target has to be included in any therapeutic approach in septic shock resuscitation.

A therapeutic strategy combining volume resuscitation, use of vasopressors, inotropic agents, vasodilators, and RBC transfusions (aimed to obtain an adequate global oxygen delivery) will not succeed in improving the outcome if it cannot recruit the microcirculation nor restore the microvascular flow [37]. 

Dubin et al. demonstrated in twenty septic shock patients that reaching a good MAP (>65 mmHg) with increasing doses of norepinephrine can improve cardiac index, pulmonary pressures, systemic vascular resistance, and left and right ventricular stroke work indexes, but not the microvascular perfusion. It might be harmful in some patients [38]. 

Sakr et al., as well as many other authors, demonstrated that the recovery of macrohemodynamic stability does not necessarily match with microhemodynamic improvement, organ function restoration, and improved survival; experimental models of resuscitated septic shock show that microvascular perfusion is often altered despite the normalization of systemic and regional hemodynamic [39].

Bateman and Walley showed that microhemodynamic restoration leads to organ function improvement and evident decrease in mortality [40].

Furthermore, according to Top et al., persistent microcirculatory alteration can be prognostic of mortality [41].

Therefore, blood pressure, cardiac index, and other macrohaemodynamical variables have not to be considered as reliable markers of septic shock recovery.

De Backer et al. showed no correlation between arterial blood pressure and microvascular perfusion during sepsis, while they demonstrated the relation between the proportion of perfused capillaries and mortality [42]. Similar results were then reported on a larger sample by Sakr et al. [43].

In the last years, many researchers focused their attention on the microvascular response to pharmacological interventions, in an attempt to find therapies able to restore the microcirculatory flow. 

The activated-protein C (aPC) is among the most interesting and studied drugs [44]; a nonrandomized study, conducted by de Backer et al., have demonstrated that an infusion of aPC in septic patients can improve the microcirculatory flow. An increase in the proportion of perfused capillaries and a more rapid resolution of hyperlactatemia have been also found in the aPC treated patients, unlike the control group [45].

In a prospective observational open study, Donati et al. measured the tissue oxygen saturation (StO2) using the near infrared spectroscopy during a vascular occlusion test and demonstrated an improvement in both the StO2 downslope and upslope in patients treated with aPC, unlike the controls, reflecting an improved microvascular reactivity [46].

An aPC administration during experimental endotoxaemia can improve intestinal microcirculation by protecting functional capillary density and exerts an anti-inflammatory effect by reducing leukocyte rolling and adherence to the endothelium in each submucosal venule; protection from leukocytic inflammation is probably mediated by a modulation of adhesion molecules expression on the surface of leukocytes and endothelial cells [47].

Unfortunately, aPC was removed from clinical use by the company after the PROWESS-Shock trial in septic shock patients because it failed to reduce mortality, compared to placebo [48].

Morelli et al. obtained good results using terlipressin (a vasopressin analogue, relatively selective for V1 receptors) as adjunctive vasopressor agent in experimental models of vasodilatatori hyperdynamic septic shock unresponsive to catecholamine infusion; both bolus and continuous infusion of terlipressin seem to improve the microcirculation [49–51].

It is not clearly understood whether a continuous or intermittent infusion is to be preferred; recent studies demonstrated that a continuous therapy is associated with less organ dysfunction in endotoxemic sheep, but the relationship with septic shock outcome is not clear [52].

Finally it was recently demonstrated that levosimendan is better than dobutamine in improving the microcirculation in stabilized septic shock patients [53].

## 3. Microcirculation in Cardiac Failure

Few years after these studies many authors turn their attention to the microvascular reactions to acute heath failure (AHF) and cardiogenic shock.

De Backer et al. evaluated the sublingual microcirculation in 40 patients within the first 48 hours after an AHF and found alterations similar to those of sepsis; while the capillary density and the perfusion of large vessels were preserved, the proportion of perfused small vessels (PPVs) was acutely reduced and the extent of such reduction strictly related to survival [54]. An example of sublingual microcirculation derangement during cardiogenic shock can be seen in Figure 1(d).

The reliability of these results is so strong that the sublingual SDF was used by Lam et al. to evaluate the effective myocardial recovery and the optimization of organ perfusion in STEMI patients treated with PCI and a percutaneous left ventricular support [55].

The main difference between cardiogenic shock and septic microvascular derangement is that microvascular alterations in AHF are not completely independent from changes in macrocirculation; indeed, a relationship between cardiac output and microcirculatory status can be seen.

Therefore, a good therapeutic strategy should target restoring both macro- and microcirculation. Many clinical approaches have been considered, aiming to evaluate which one fulfills both objectives. For example, Erol-Yilmaz et al. proved that the cardiac resynchronization therapy (CRT) used to improve the systemic pressure can stabilize also the microcirculation [56]. Additionally, Munsterman et al. demonstrated that the intra-aortic balloon pump used to mechanically support the hearth often impairs the microcirculatory flow and its withdrawal can paradoxically improve the microcirculatory flow of small vessels [57].

Besides, den Uil et al. used the SDF imaging in patients with AHF during and after a low dose administration on nitroglycerin (NTG = 33 *μ*g/min⁡  IV), which is sufficient to decrease central venous pressure and pulmonary capillary wedge pressure. They found a significant increase of PCD in patients responding to NTG; therefore, NTG does affect not only uniquely the cardiac muscle but also any peripheral tissue [58].

Future studies might examine the response to higher doses of NTG, nitroprusside (which releases NO by non-enzymatic means), and hydralazine (a nonNO donor vasodilator) in order to better understand the situation of nonrespondant patients.

## 4. Microcirculation in Hypovolemia

Functional capillary density deteriorates in hypovolemia when the mean blood pressure drastically decreases.

The ultimate goal of volume replacement therapy is to improve organ perfusion thereby sustaining an adequate oxygenation. Too few studies have been conducted to evaluate the effects of this strategy on microcirculation, but colloids generally tend to be considered superior to crystalloids in improving tissue perfusion [59]. However, whether they can also improve the outcomes still needs to be proved.

Microcirculatory changes are smaller in hypovolemia than sepsis; for similar blood pressure levels, hypovolemic rats showed a lower percentage of nonperfused capillaries than septic shock rats, and the red blood cell velocity was nearly always preserved. Even if the observed alterations are fewer than those in sepsis, they are though related to mortality in animal models [60]. A typical example of sublingual microcirculation during hypovolemia is provided in Figure 1(c).

Hemorrhagic shock leads to intestinal microvascular endothelium damage; the endothelial cells become oedematous and cell membrane and mitochondria are quickly injured; SOD activity is enhanced and the activity of CAT and GSH-PX decreases. Korzonek and Gwóźdź proved that an I.V. administration of endothelin-1 can restore normal blood pressure, prevent it to fall, and restrict the ischemic injury on microcirculation, thereby prolonging the survival in animals with hemorrhagic shock [61].

In addition, the results presented by Fang et al. suggest that in hypovolemia, as well as cardiac failure, microvascular alterations are not completely independent from global haemodynamic parameters [62]. 

An experimental study by Legrand et al. showed that kidneys are particularly prone to hypoxia even in a high MAP-directed fluid resuscitation (>80 mmHg); the renal microvascular PO2 does not improve compared with fluid resuscitation targeting to a MAP = 40 mmHg. Moreover, a decreased renal oxygenation persists after blood transfusion [63].

These findings need to be confirmed in human studies and resuscitation strategy for hemorrhagic shock remains controversial.

## 5. Conclusions

Further research is required to improve microcirculatory flow knowledge. A recent multicenter prevalence study [64] is aimed to assess the relationship between microcirculatory dysfunction and severity of illness and to investigate the prevalence of sublingual microcirculatory alterations in intensive care unit (ICU) patients, regardless of their underlying disease, monitored at a single time point in all the different participating centers. This is the first step towards a more comprehensively understanding of what happens at the microcirculatory level during life-threatening illness, to identify the relationship with macrohemodynamics and to evaluate whether drugs used in ICU to improve hemodynamic status and organ functions can also improve the microcircuvascular flow. The biggest step forward will be made when treatments selectively targeted to resuscitate the microcirculation will be found.

For the moment, according to our knowledge, we can state: treat the macrohemodynamic as soon as possible, but if the patient does not get better, look at the microcirculation and try to resuscitate it!

## Figures and Tables

**Table 1:** Parameters for the evaluation and scoring of the microcirculation.

Microcirculation parameter	Information provided	Measurement
Microvascular flow index (MFI)	Perfusion quality (for small, medium, and large vessels*)	The image is divided into four quadrants; a number is assigned for each quadrant according to the predominant type of flow (0 = no flow; 1 = intermittent; 2 = sluggish; 3 = continuous). The MFI results from the averaged values.

De Backer score (n/mm)	Vessel density	The image is divided by 3 vertical and 3 horizontal lines; the De Backer score is calculated as the number of vessels crossing the lines divided by the total length of the lines

Total vessel density (mm/mm^2^)	Vessel density (for small, medium, and large vessels*)	Total length of vessels is divided by the total surface of the analyzed area

Perfused vessel density (mm/mm^2^)	Functional vessel density (for small, medium, and large vessels*)	Total length of perfused vessels (sluggish or continuous) is divided by the total surface of the analyzed area

Proportion of perfused vessels (%)	Perfusion quality (for small, medium, and large vessels*)	100* number of perfused vessels is divided by the total number of vessels

Flow heterogeneity index (FHI)	Perfusion heterogeneity	The difference between the highest MFI and the lowest MFI is divided by the mean MFI. MFI is intended as the averaged MFI of each site

*Vessel diameter classification: <20 *μ* = small; 20–50 *μ* = medium; 50–100 *μ* = large.

Three or five sites are evaluated. MFI of small vessels can be calculated separately.
